# Hydroquinones Inhibit Biofilm Formation and Virulence Factor Production in *Staphylococcus aureus*

**DOI:** 10.3390/ijms231810683

**Published:** 2022-09-14

**Authors:** Sanghun Kim, Jin-Hyung Lee, Yong-Guy Kim, Yulong Tan, Jintae Lee

**Affiliations:** 1 School of Chemical Engineering, Yeungnam University, Gyeongsan 38541, Korea; 2 Special Food Research Institute, Qingdao Agricultural University, Qingdao 266109, China

**Keywords:** biofilms, hydroquinones, *RNAIII*, *Staphylococcus* *aureus*, virulence factors

## Abstract

*Staphylococcus aureus* is one of the major pathogens responsible for antimicrobial resistance-associated death. *S. aureus* can secrete various exotoxins, and staphylococcal biofilms play critical roles in antibiotic tolerance and the persistence of chronic infections. Here, we investigated the inhibitory effects of 18 hydroquinones on biofilm formation and virulence factor production by *S. aureus*. It was found that 2,5-bis(1,1,3,3-tetramethylbutyl) hydroquinone (TBHQ) at 1 µg/mL efficiently inhibits biofilm formation by two methicillin-sensitive and two methicillin-resistant *S. aureus* strains with MICs of 5 µg/mL, whereas the backbone compound hydroquinone did not (MIC > 400 µg/mL). In addition, 2,3-dimethylhydroquinone and *tert*-butylhydroquinone at 50 µg/mL also exhibited antibiofilm activity. TBHQ at 1 µg/mL significantly decreased the hemolytic effect and lipase production by *S. aureus*, and at 5–50 µg/mL was non-toxic to the nematode *Caenorhabditis elegans* and did not adversely affect *Brassica rapa* seed germination or growth. Transcriptional analyses showed that TBHQ suppressed the expression of *RNAIII* (effector of quorum sensing). These results suggest that hydroquinones, particularly TBHQ, are potentially useful for inhibiting *S. aureus* biofilm formation and virulence.

## 1. Introduction

Methicillin-, vancomycin-, and multidrug-resistant strains of *Staphylococcus aureus* are human pathogens responsible for hospital-acquired infections with high morbidities and mortalities. *S. aureus* produces many exotoxins that contribute to its ability to colonize skin and nasal mucosa and cause diseases in mammalian hosts [1]. Furthermore, its ability to form biofilms on medical devices and host surfaces is of particular concern because biofilm formation is associated with resistance to conventional antibiotics and host defense systems [2,3]. Hence, targeting biofilm formation and/or toxin production are considered alternative means of controlling drug-resistant *S. aureus* infections.

Most *S. aureus* strains produce cytotoxins and enzymes such as hemolysins, nucleases, proteases, lipases, hyaluronidase, and collagenase that convert local host tissues into nutrients required for *S. aureus* growth [1]. Additionally, the bacterium has several host immune evasion strategies, which include staphyloxanthin production and biofilm formation. Hence, novel compounds that inhibit biofilm formation and virulence factor production are urgently required, and importantly, these compounds must not enhance drug-induced evolutionary pressure toward the development of drug resistance.

Numerous natural and synthetic compounds have been reported to inhibit biofilm formation and toxin production by *S. aureus*. In particular, several anthraquinones (e.g., alizarin, purpurin, emodin, quinalizarin, and hydroxyanthraquinones) have been reported to inhibit biofilm formation by *S. aureus* [4,5,6], whereas the structural backbones of these molecules (hydroquinone and anthraquinone) have no or little effect on *S. aureus* biofilm formation [5]. Hence, this study was undertaken to identify novel hydroquinones exhibiting antimicrobial, antibiofilm, and antivirulence activities against methicillin-sensitive *S. aureus* (MSSA) and methicillin-resistant *S. aureus* (MRSA) strains and to investigate the mechanisms responsible. Live imaging microscopy, confocal laser scanning microscopy (CLSM), and scanning electron microscopy (SEM) were used to examine their antibiofilm effects. Hemolysis, lipase, and staphyloxanthin assays and quantitative real-time reverse transcription polymerase chain reaction (qRT-PCR) were used to elucidate the molecular basis of their activities, and plant seed germination and nematode models were used to investigate their toxicities.

## 2. Results

### 2.1. Antibiofilm and Antimicrobial Activities of Hydroquinone (HQ) and the 17 HQ Derivatives against S. aureus

**Figure 1:**
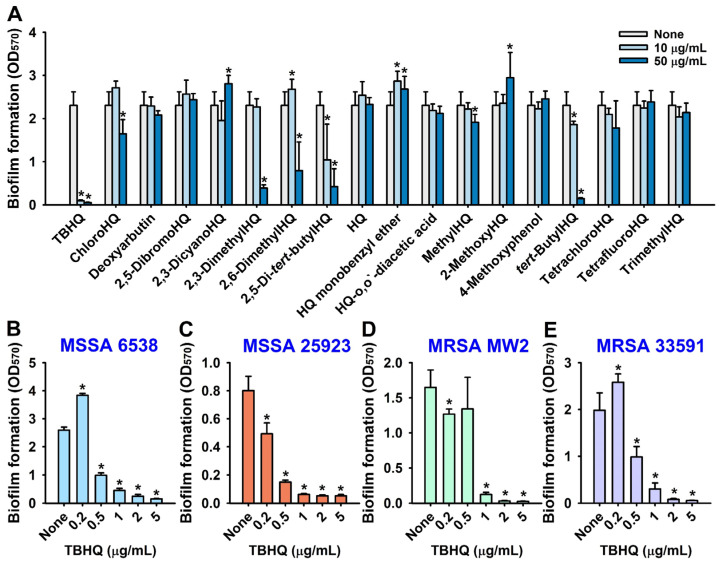
Inhibition of *S. aureus* biofilm formation by hydroquinones. The antibiofilm activities of HQ and its derivatives on MSSA 6538 were investigated in 96-well polystyrene plates after culture for 24 h (**A**). The antibiofilm activities of TBHQ were investigated against MSSA 6538 (**B**) and MSSA 25923 (**C**), MRSA MW2 (**D**), and MRSA 33591 (**E**) after culture for 24 h. * *p* < 0.05 vs. non-treated controls (None).

The biofilm inhibitory effects of the 18 hydroquinones at 10 or 50 µg/mL were initially investigated against the MSSA 6538 strain in 96-well polystyrene plates. The antibiofilm efficacies of the 18 hydroquinones differed widely (Figure 1A). Three HQ derivatives, namely, 2,5-bis(1,1,3,3-tetramethylbutyl) hydroquinone (TBHQ), 2,3-dimethylhydroquinone, and *tert*-butylhydroquinone at 50 µg/mL exhibited significant antibiofilm activity. Notably, TBHQ at 10 or 50 µg/mL inhibited *S. aureus* biofilm formation by >96%.

The antimicrobial activities of the 18 hydroquinones were investigated by measuring minimum inhibitory concentrations (MICs). TBHQ, 2,3-dimethylhydroquinone, and 2,6-dimethylhydroquinone had MICs of 5, 50, and 50 µg/mL, respectively, whereas most other hydroquinones had MICs of >400 µg/mL and hydroquinones have an MIC of 100 or 200 (Table 1). Accordingly, due to its potent antimicrobial and antibiofilm activities, TBHQ was selected for further assays, and HQ was used as a structural control.

A more detailed biofilm study showed that TBHQ at lower doses (0.2, 0.5, 1, 2, or 5 µg/mL) dose-dependently inhibited biofilm formation by the two methicillin-sensitive *S. aureus* strains (MSSA 6538 and MSSA 25923) and two methicillin-resistant *S. aureus* strains (MRSA MW2 and MRSA 33591) (Figure 1B–E). For example, at 1 or 2 µg/mL, TBHQ inhibited *S. aureus* biofilm formation by all four strains by ~93% and ~95%, respectively, with MICs of ~5 µg/mL. Interestingly, at the subinhibitory concentration of 0.2 µg/mL, TBHQ increased biofilm formation by 48% and 30% against MSSA 6538 and MRSA 33591, respectively, as compared with non-treated controls. This phenomenon is in line with a previous report that subinhibitory concentrations of aminoglycoside antibiotics induced biofilm formation by *Pseudomonas aeruginosa* and *Escherichia coli* [7]. Furthermore, planktonic cell growth curves confirmed that the MIC of TBHQ was 5 µg/mL and that at 1 to 2 µg/mL TBHQ delayed planktonic cell growth, whereas HQ had no effect (Figure S1). These results indicate that the antimicrobial activity of TBHQ partially contributed to its inhibition of *S. aureus* biofilm formation.

### 2.2. Microscopic Observations of S. aureus Biofilm Inhibition by TBHQ

**Figure 2:**
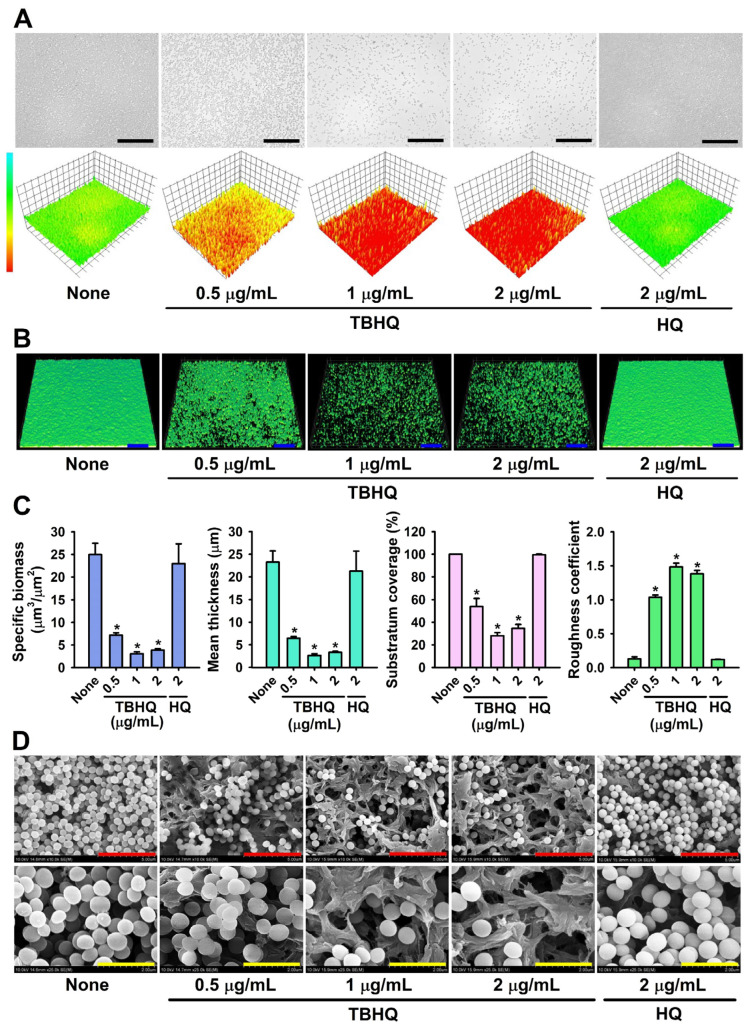
Microscopic observations of *S. aureus* biofilm inhibition by TBHQ. Constructed color-coded 3D images of MSSA 6538 biofilms after culture with TBHQ or HQ (**A**), CLSM images (**B**) and COMSTAT analysis (**C**), SEM analysis (**D**). Error bars represent standard deviations. * *p* < 0.05 vs. non-treated controls (None). The black, blue, red, and yellow scale bars represent 50, 100, 5, and 2 μm, respectively.

Bright-field microscopy, CLSM, and SEM were used to observe biofilm inhibition by TBHQ. For non-treated biofilms, 3D color mesh plots were green, indicating abundant biofilm formation, whereas TBHQ at 0.5–2 µg/mL produced yellow to red plots, indicating poor or no biofilm formation. Treatment with HQ had no effect on color plots (Figure 2A). CLSM also showed that TBHQ dose-dependently inhibited biofilm formation vs. untreated controls (Figure 2B). Biofilm reduction was further quantified using COMSTAT biofilm software, which showed that TBHQ at 1 or 2 μg/mL dramatically reduced average biofilm thickness and substratum coverage and increased biofilm roughness (Figure 2C). Specific biomass, mean thickness, and substratum coverage were reduced by TBHQ at 1 μg/mL by 88%, 89%, and 72%, respectively, vs. non-treated controls. SEM analysis (Figure 2D) also showed that TBHQ markedly reduced the numbers of *S. aureus* cells, but only slightly affected *S. aureus* cell morphology on nylon membranes at concentrations of 2 μg/mL. These microscopic findings confirmed that TBHQ at subinhibitory concentrations dose-dependently inhibited *S. aureus* biofilm formation.

### 2.3. Inhibitory Effects of TBHQ on Virulence Factors in S. aureus

**Figure 3:**
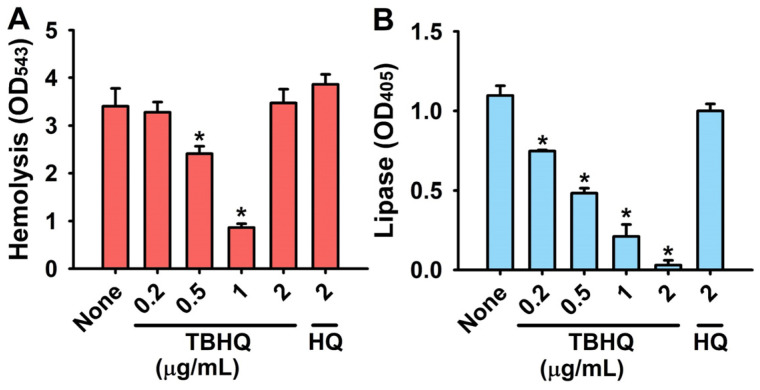
Inhibitory effects of TBHQ on virulence factors in *S. aureus*. Anti-hemolytic activity (**A**) and extracellular lipase production (**B**). MSSA 6538 was used for these assays. Error bars indicate standard deviations. * *p* < 0.05 vs. non-treated controls (None).

We used hemolysis, lipase, and staphyloxanthin assays to investigate how TBHQ exerts its antimicrobial and antibiofilm activities. *S. aureus* produces *α*-toxin, which causes hemolysis and is required for *S. aureus* biofilm formation [8] and thus, we investigated the effect of TBHQ on the hemolytic activity of *S. aureus*. Treatment of TBHQ at 0.2–1 µg/mL dose-dependently inhibited hemolytic activity, while HQ at 2 µg/mL had no effect (Figure 3A).

Lipase activities were measured because staphylococcal lipases have been shown to promote biofilm formation and host cell invasion [9]. TBHQ dose-dependently inhibited the activity of extracellular lipases at 0.2–2 µg/mL in *S. aureus*, but HQ had no effect (Figure 3B).

*S. aureus* also produces staphyloxanthin (an antioxidant and important virulence factor) as a countermeasure to host immune defense systems [10]. However, TBHQ and HQ at concentrations ≤2 µg/mL did not appear to reduce the production of staphyloxanthin as determined by the yellow color of cell pellets.

### 2.4. TBHQ Repressed the Expressions of Quorum Sensing Gene RNAIII

**Figure 4:**
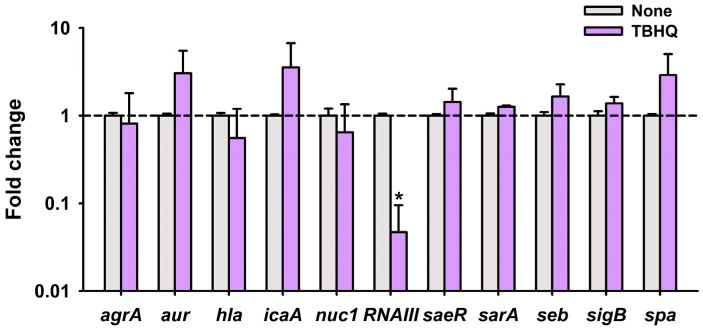
Transcriptional profiles of *S. aureus* cells treated with or without TBHQ. MSSA 6538 was cultivated for 3 h with shaking at 250 rpm and then incubated with or without TBHQ (1 μg/mL) for 4 h with shaking at 250 rpm. Transcriptional profiles were measured by qRT-PCR. Fold change represents transcriptional changes vs. non-treated *S. aureus*. The experiment was performed in duplicate. * *p* < 0.05 vs. non-treated controls (None).

qRT-PCR was used to examine the differential expressions of 11 biofilm- and toxin-related genes after treating *S. aureus* with TBHQ at 1 μg/mL for 4 h. Interestingly, TBHQ reduced the expression of *RNAIII* (effector of quorum sensing) by 21-fold but had a lesser effect on the expression of other genes in this condition (Figure 4). Since it has been well-established that quorum sensing positively controls virulence attributes and biofilm formation, this qRT-PCR result suggests that TBHQ inhibits biofilm formation and virulence factor production via downregulating the expression of *RNAIII.*

### 2.5. Toxicity of Hydroquinones in the Plant Germination and Nematode Models

**Figure 5:**
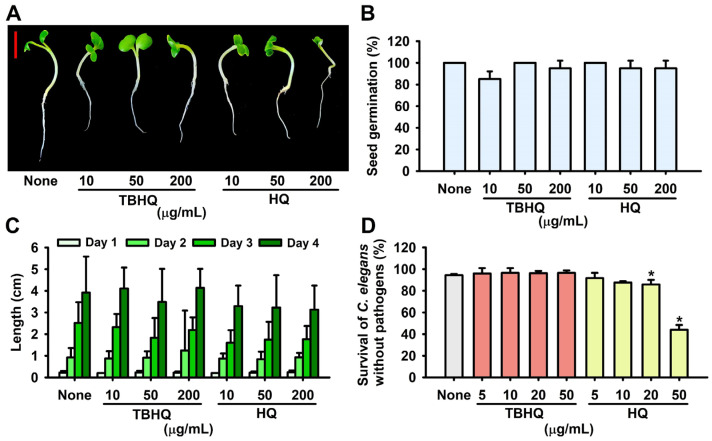
Toxicities of TBHQ and HQ in the plant and nematode models. *B. rapa* growth and seed germination was performed using Murashige and Skoog agar medium supplemented with or without TBHQ or HQ at 25 °C (**A**). Seed germination rates (**B**) and plant lengths (**C**) were measured over 4 days. *C. elegans* survival was assessed in the presence or absence of TBHQ or HQ after 4 days (**D**). * *p* < 0.05 vs. non-treated controls (None). The red scale bar represents 1 cm.

Since the safety and toxicity of hydroquinone have been questioned [11], toxicity assessments of TBHQ and HQ were conducted using a *Brassica rapa* seed germination and a *Caenorhabditis elegans* nematode model. Plant growth was similar for 4 days after TBHQ or HQ treatment (Figure 5A,C), and the seed germination rate was unaffected by TBHQ or HQ at 10, 50, or 200 μg/mL (Figure 5B). In the *C. elegans* model, TBHQ was less toxic than HQ (Figure 5D). For example, most nematodes survived after treatment with TBHQ at 50 μg/mL for 4 days, whereas 44% of worms died after treatment with HQ under the same conditions. These results indicate that TBHQ is not toxic to *B*. *rapa* or *C. elegans* in its active antibiofilm range (1–2 μg/mL).

### 2.6. ADME Profiling of TBHQ

ADME (absorption, distribution, metabolism, and excretion) profiles of TBHQ were also evaluated. TBHQ did not violate Lipinski’s rule of five [12], had acceptable skin and brain barrier permeabilities and human intestinal adsorptions, did not exhibit acute fish toxicity, and was non-carcinogenic to mice. Full ADME profiles are presented in Table S1.

## 3. Discussion

We report the antimicrobial and antibiofilm abilities of a series of hydroquinones against *S. aureus*. The most active hydroquinone, TBHQ, at subinhibitory concentrations (0.5–1 µg/mL), significantly inhibited biofilm formation, hemolytic activity, and extracellular lipase production. Notably, TBHQ significantly suppressed the expression of *RNAIII* and exhibited no toxicity in the plant growth and nematode models.

Hydroquinone (HQ) has been used as an anti-hyperpigmentation agent for more than 40 years [11], and HQ at 300–2000 µg/mL has been reported to exhibit antimicrobial activities against *S. aureus* strains, probably by targeting cell walls and membrane [13,14], but the molecular mechanism involved has not been elucidated. This study shows that TBHQ at 1–5 µg/mL exhibits potent antibiofilm and antibacterial activities against *S. aureus* strains (Table 1 and Figure 1) and is less toxic than HQ (Figure 5). Toxicology of hydroquinone and *tert*-butylhydroquinone were reported as these hydroquinones at high concentrations induced a process of carcinogenesis [11,15]. Since there is no toxicological report of TBHQ yet, more rigorous toxicity assessment is required, and drug carrier systems could be utilized to slowly release the active compound and to reduce cell toxicity.

Notably, qRT-PCR demonstrated that TBHQ at 1 µg/mL significantly repressed the expression of quorum sensing-related *RNAIII* but not those of other biofilm-related genes (Figure 4). *RNAIII* is a regulatory RNA molecule that binds to quorum sensing accessory gene regulator A (*agrA*) [16] and positively regulates hemolysins, lipases, exoproteases, enterotoxins, and the methicillin resistance of *S. aureus* [17]. Hence, it appears that TBHQ inhibited virulence factor production (Figure 3) by repressing *RNAIII* expression. Since many small molecules or peptides bind to AgrA or RNAIII [18], it would be interesting to investigate whether and how TBHQ binds to AgrA and/or RNAIII.

The *agr* and *RNAIII* system controls *S. aureus* biofilm development [19], and several peptides that inhibit *RNAIII* also inhibit *S. aureus* biofilm formation [20,21]. Furthermore, *α*-hemolysin and lipase, which are both regulated by *RNAIII,* play positive roles in *S. aureus* biofilm formation [8,9]. We observed that TBHQ decreased hemolytic and lipase activity (Figure 3), which supports its observed inhibitory effect on *S. aureus* biofilm formation. Taken together, these observations indicate that TBHQ inhibits biofilm formation and virulence factor production by downregulating *RNAIII*.

Several hydroquinone derivatives such as methylhydroquinone [22], methyl-1,4-hydroquinone, 2,3-dimethyl-1,4-hydroquinone [23], *tert*-butylhydroquinone [24], and trimethylhydroquinone [25] have been reported to inhibit *S. au*reus cell growth, but this is the first time that TBHQ has been reported to exhibit antibacterial, antibiofilm, and anti-toxin activities against *S. aureus*. Intriguingly, 2,5-di-*tert*-butylhydroquinone and *tert*-butylhydroquinone exhibited low antibacterial activities against *S. aureus* with MICs of ≥200 µg/mL (Table 1). TBHQ differs from 2,5-di-*tert*-butylhydroquinone by the addition of two additional *tert*-butyl groups, which indicates that the 2,5-tetramethylbutyl groups in TBHQ crucially influence antimicrobial and antibiofilm activities against *S. aureus*.

Overuse of antibiotics has led to the development of drug-resistant *S. aureus* strains. In order to address drug-resistant *S. aureus* infections properly, anti-virulence approaches based on, for example, inhibitions of toxin production, biofilm formation, and quorum sensing, offer potential therapeutic strategies [26,27,28]. The current study demonstrates that TBHQ exhibits antibacterial, antibiofilm, and anti-toxin activities against *S. aureus* with low toxicity and thus identifies TBHQ as a potential non-toxic, antivirulence compound against recalcitrant *S. aureus* infections. Further molecular and in vivo studies are required to provide more detail on the molecular mechanisms involved and to identify possible applications.

## 4. Materials and Methods

### 4.1. Bacterial Strains, Growth Measurements, and Materials

Two MSSA strains (ATCC 25923 and ATCC 6538) and two MRSA strains (MW2 and ATCC 33591) were used in the study. Experiments were conducted at 37 °C in LB medium for the two MSSA strains and in LB medium containing 0.2% glucose for the MRSA MW2 and 33591 strains. To assess cell growths, culture turbidities were measured at 620 nm using a spectrophotometer (Multiskan EX microplate reader; Thermo Fisher Scientific, Waltham, MA, USA) after cultivation for 24 h at 37 °C. The 18 hydroquinone compounds tested were as follows: 2,5-bis(1,1,3,3-tetramethylbutyl) hydroquinone, chlorohydroquinone, deoxyarbutin, 2,5-dibromohydroquinone, 2,3-dicyanohydroquinone, 2,3-dimethylhydroquinone, 2,6-dimethylhydroquinone, 2,5-di-*tert*-butylhydroquinone, hydroquinone, hydroquinone monobenzyl ether, hydroquinone-*O*,*O*′-diacetic acid, methylhydroquinone, 2-methoxyhydroquinone, 4-methoxyphenol, *tert*-butylhydroquinone, tetrachlorohydroquinone, tetrafluorohydroquinone, and trimethylhydroquinone (Table 1). All were purchased from Sigma-Aldrich (St. Louis, MO, USA) or Combi-blocks (San Diego, CA, USA) and dissolved in dimethyl sulfoxide (DMSO), which did not exceed 0.1% (*v/v*) in any experiment. For MIC experiments, *S. aureus* cells (10^7^ cells/mL) were cultured overnight in LB medium, added to the wells of 96-well polystyrene plates (SPL Life Sciences, Pocheon, Korea) containing different concentrations (*w/v*) of the 18 hydroquinone derivatives, and incubated for 24 h at 37 °C without shaking. To assess planktonic cell growths, culture turbidities were measured at 600 nm using a spectrophotometer (Optizen 2120 UV; Mecasys Co., Ltd., Daejeon, South Korea) after cultivation in 250 mL flasks for 24 h at 37 °C. All experiments were performed using at least two independent cultures.

### 4.2. Crystal-Violet Biofilm Assay

A static biofilm formation assay was performed in 96-well polystyrene plates, as previously reported [29]. Briefly, cells were inoculated into LB medium (300 µL) at an initial turbidity of 0.05 at 600 nm (10^7^ cells/mL) and cultured with or without hydroquinones for 24 h without shaking at 37 °C. To quantify biofilm formation, biofilms were stained with crystal violet, dissolved in 95% ethanol, and absorbances were measured at 570 nm (OD_570_). Cell growths in 96-well plates were also measured at 620 nm (OD_620_). Biofilm formation and static cell growth results are presented as the means of at least six replicate wells.

### 4.3. Observations of Biofilm Inhibition by Live Imaging Microscopy, CLSM, and SEM

Biofilms were produced, as mentioned above, over 24 h at 37 °C. Free-floating cells were then removed by gentle washing with distilled H_2_O three times, and biofilms were visualized by live imaging microscopy using the iRiS™ Digital Cell Imaging System (Logos Bio Systems, Anyang, Korea). Biofilm images were generated as color-coded 3D pictures using ImageJ (https://imagej.nih.gov/ij/index.html, accessed on 22 May 2022). In addition, cells were cultivated in 96-well polystyrene plates without shaking in the absence or presence of hydroquinones. Free-floating cells were then removed by washing with sterile PBS buffer three times, and biofilms were incubated with carboxyfluorescein diacetate succinimidyl ester (a cell-permeable dye; Thermo Fisher Scientific, Waltham, MA, USA) [30], which becomes fluorescent when it loses its acetyl groups due to intracellular esterase activity. Biofilms were then visualized at 488 nm using an Ar laser (emission wavelength 500 to 550 nm) confocal laser scanning microscope (Nikon Eclipse Ti, Tokyo, Japan) equipped with a 20× objective [31]. Color confocal images were constructed using NIS-Elements C version 3.2 (Nikon Eclipse). At least 10 random positions in two independent cultures were analyzed per experiment. To quantify biofilm formation, color confocal images (20 image stacks) were converted to gray scale using ImageJ. COMSTAT biofilm software was used to determine biomasses (μm^3^ per μm^2^), mean biofilm thicknesses (μm), substratum coverages (%), and roughness coefficients. Thresholding was fixed for all image stacks, and at least four positions and 20 planar images per position were analyzed. SEM was also performed, as previously described [32]. Briefly, a sterile nylon membrane (Whatman, Maidstone, UK) was cut into 0.4 × 0.4 cm pieces, and then single pieces were set in the wells of 96-well plates containing 300 µL of cell suspension of turbidity 0.1 at 600 nm. Cells were incubated in the presence or absence (untreated controls) of hydroquinones for 24 h at 37 °C without shaking. Biofilm cells were then fixed with a glutaraldehyde (2.5%) and formaldehyde (2%) mixture for 24 h at 4 °C, post-fixed in osmium tetroxide (1% OsO_4_ solution), and dehydrated using an ethanol series (50, 70, 80, 90, 95, and 100%) followed by isoamyl acetate. After critical-point drying, cells on membranes were examined under an S-4800 field emission scanning electron microscope (FE-SEM, Hitachi, Tokyo, Japan) at a voltage of 10 kV and magnifications ranging from ×10,000 to ×25,000.

### 4.4. Hemolysis Assay

Sheep red blood cell hemolysis efficacies were assessed using whole cultures of *S. aureus*, as described previously [33,34]. Briefly, MSSA 6538 cells were diluted 1:100 in LB medium and cultured with or without hydroquinones (0, 0.2, 0.5, 1, or 2 µg/mL) for 24 h at 250 rpm. Fresh whole sheep blood was purchased from MBcell (Seoul, Korea) and red blood cells were obtained by centrifuging the whole sheep blood at 3000 rpm for 5 min, washing cells with sterile PBS five times, and then diluted in PBS (330 µL of red blood cells in 10 mL of PBS). MSSA 6538 cultures (300 µL) were mixed with 1 mL of the diluted red blood cells. To determine hemolytic activities, mixtures of red blood and MSSA 6538 (300 µL of cell culture) were incubated at 250 rpm for 3 h at 37 °C. Supernatants were collected by centrifugation at 10,000 rpm for 10 min, and optical densities were measured at 543 nm.

### 4.5. Lipase Production Assay

To investigate the effect of hydroquinones on extracellular lipase production, MSSA 6538 cells were diluted in LB medium at 1:100 and incubated for 24 h at 37 °C with or without hydroquinones (0, 0.2, 0.5, 1, or 2 µg/mL). Supernatants were then collected by centrifugation at 8000× *g* for 10 min, and 0.1 mL aliquots were mixed with 900 µL of substrate buffer (1 part (by vol.) of buffer A (3 mg/mL of *p*-nitrophenyl palmitate in 2-propanol) and 9 parts (by vol.) of buffer B (1 mg/mL of gummi arabicum and 2 mg/mL sodium deoxycholate in 0.05 M disodium hydrogen phosphate buffer (pH 8.0)) at 25 °C for 30 min in the dark. Lipase reactions were stopped by adding 0.1 mL of 1 M sodium carbonate. Absorbances of supernatants were measured at 405 nm as previously described [35]. Two independent samples were analyzed.

### 4.6. RNA Isolation

For qRT-PCR experiments, the RNAs of MSSA 6538 cells were isolated using the following procedure. MSSA 6538 cells (10^7^ cells/mL) were inoculated into 25 mL of LB medium at 37 °C in 250 mL shake flasks with overnight cultures (1: 100 dilution) and cultured for 3 h with shaking at 250 rpm. TBHQ (1 µg/mL) was then added, and incubation was continued for a further 4 h. Before sample collection, RNase inhibitor (Ambion, TX, USA) was added, and cells were centrifuged at 10,000 rpm for 2 min. Cell pellets were immediately frozen in dry ice and stored at −80 °C. RNA was isolated using the Qiagen RNeasy mini Kit (Valencia, CA, USA). RNA quality was assessed using a NanoVue Plus (Biochrom Ltd., Cambridge, UK).

### 4.7. qRT-PCR

qRT-PCR was used to determine the relative transcription levels of important biofilm-related genes (*agrA*, *aur*, *hla*, *icaA*, *nuc1*, *RNAIII*, *saeR*, *sarA*, *seb*, *sigB*, and *spa*) in MSSA 6538 cells. Gene-specific primers were used (Table S2), and the expression level of *16s rRNA* (housekeeping control) was used to normalize the expressions of genes of interest. The qRT-PCR method used was adapted from a previous study [35]. PCR was performed using a SYBR Green master mix (Applied Biosystems, Foster City, CA, USA) and an ABI StepOne Real-Time PCR system (Applied Biosystems). Expression levels were determined using two independent cultures, which produced similar results.

### 4.8. Seed Germination Assay

The effects of hydroquinones on plant seed germination were assessed using Murashige and Skoog agar plates, as previously described [36]. Briefly, *B. rapa* seeds were washed five times with sterile distilled water, soaked in sterile distilled water for 24 h, rinsed several times with sterilized distilled water, and carefully placed on agar plates containing 0.86 g/L Murashige and Skoog medium supplemented with 0.7% bacto-agar and hydroquinones at 10, 50, or 200 µg/mL. Plates were then incubated at 25 °C, and images were captured after incubation for 4 days. Germination percentages and plant heights were recorded.

### 4.9. Cytotoxicity Assays

Non-infected nematodes (*C. elegans*) were pipetted into the wells of 96-well plates (30 nematodes/well) containing M9 buffer and hydroquinones at concentrations of 0, 5, 10, 20, or 50 µg/mL. Plates were then incubated for 4 days at 25 °C without shaking. Results are expressed as percentage survivals. Observations were made using an iRiS™ Digital Cell Imaging System according to the manufacturer’s instructions.

### 4.10. Statistical Analysis

The analysis was performed by one-way ANOVA followed by Dunnett’s test in SPSS Ver. 23 (Chicago, IL, USA). Results are presented as means ± SDs, and *p* values of <0.05 were considered significant.

## Figures and Tables

**Table 1:** Antimicrobial activities of the 18 hydroquinones against *S. aureus*. Minimal inhibitory concentrations (MICs) and planktonic cell growth of MSSA 6538 were determined after incubation for 24 h in 96-well plates. Functional groups in HQ structures are indicated in red.

Hydroquinones	Structures	MIC	Growth (%)	
			10	50
			µg/mL	
2,5-Bis(1,1,3,3-tetramethylbutyl)hydroquinone (TBHQ)	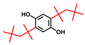	5	15	14
Chlorohydroquinone	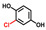	>400	82	53
Deoxyarbutin	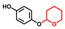	>400	96	95
2,5-Dibromohydroquinone	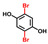	>400	86	70
2,3-Dicyanohydroquinone	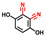	>400	95	95
2,3-Dimethylhydroquinone	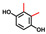	50	64	14
2,6-Dimethylhydroquinone	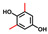	50	84	17
2,5-Di-*tert*-butylhydroquinone	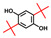	>400	78	65
Hydroquinone (HQ)	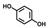	>400	93	77
Hydroquinone monobenzyl ether	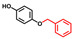	>400	101	90
Hydroquinone-*O*,*O*′-diacetic acid	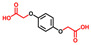	>400	96	96
Methylhydroquinone	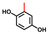	>400	90	84
2-Methoxyhydroquinone	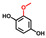	>400	86	60
4-Methoxyphenol	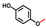	>400	92	78
*tert*-Butylhydroquinone	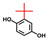	200	81	69
Tetrachlorohydroquinone	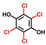	200	93	76
Tetrafluorohydroquinone	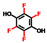	400	96	88
Trimethylhydroquinone	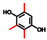	100	86	70

## Data Availability

All relevant data are provided in the manuscript.

## Supplementary Materials

The following supporting information can be downloaded at: https://www.mdpi.com/article/10.3390/ijms231810683/s1.
