# Supplementary material for: Hydroquinones Inhibit Biofilm Formation and Virulence Factor Production in Staphylococcus aureus
Source: Int J Mol Sci. 2022 Sep 14;23(18):10683. doi: 10.3390/ijms231810683 (PMC9506180; doi:10.3390/ijms231810683)
Supplement: Supplementary file 1 [file ijms-23-10683-s001.zip › ijms-1880926-supplementary.pdf]

International Journal of *Molecular Sciences*

MDPI

Article

Hydroquinones Inhibit Biofilm Formation and Virulence Factor Production in *Staphylococcus aureus*

Sanghun Kim [1,†], Jin-Hyung Lee [1,†], Yong-Guy Kim [1], Yulong Tan [2] and Jintae Lee [1,*]

[1] School of Chemical Engineering, Yeungnam University, Gyeongsan 38541, Korea
[2] Special Food Research Institute, Qingdao Agricultural University, Qingdao 266109, China
* Correspondence: jtlee@ynu.ac.kr; Tel.: +82-53-810-2533
† These authors have contributed equally to this work.

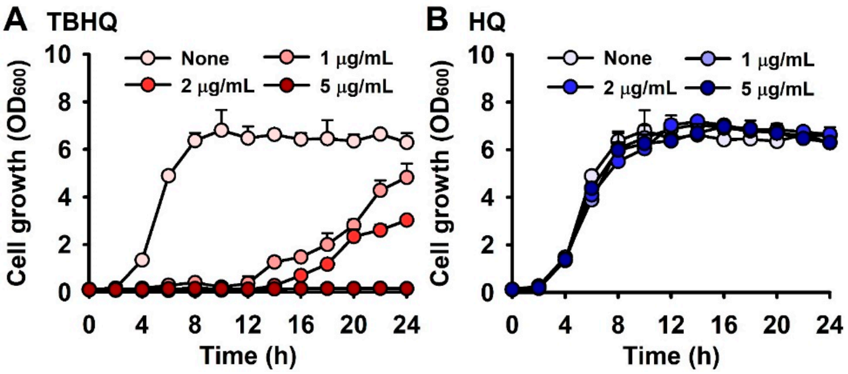

Figure S1. Planktonic cell growth in the presence of TBHQ (**A**) or HQ (**B**) against *S. aureus*. Error bars indicate standard deviations.

Table S1. ADME profiling of TBHQ and HQ.

Property	TBHQ	HQ
**Lipinski rule of five**	Suitable	Suitable
**Lipinski rule of five violations**	0	0
**Plasma protein binding**	100%	96%
**Blood brain barrier permeability**	7.64502	1.56261
**Skin permeability**	-1.0226	-3.23085
**Human intestinal absorption**	90.07%	87.42%
**Caco 2**	23.8233	16.5499
**Mouse carcinogenicity**	Negative	Negative
**Rat carcinogenicity**	Positive	Positive
**Acute algae toxicity**	0.0114243	0.107092
**Acute fish toxicity (medaka)**	0.0047614	0.573193
**Acute fish toxicity (minnow)**	0.00385119	0.174334
***In vitro* hERG inhibition**	Low	Low
**Pure water solubility**	78.667 mg/ml	45961.6 mg/ml
**miLogP**	8.02	0.98
**Mol volume**	365.31	100.08
**TPSA**	40.46	40.46
**GPCR ligand**	0.10	-3.02
**Ion channel modulator**	0.10	-2.48
**Kinase inhibitor**	-0.10	-3.07
**Nuclear receptor ligand**	0.31	-2.84
**Protease inhibitor**	-0.15	-3.20
**Enzyme inhibitor**	0.15	-2.66
**Rat IP LD50 classification**	Non-Toxic in AD	Class 4 in AD
**Rat IV LD50 classification**	Class 4 in AD	Class 4 in AD
**Rat oral LD50 classification**	Class 5 in AD	Class 4 in AD
**Rat SC LD50 classification**	Class 4 in AD	Class 4 in AD

Caco 2 - *in vitro* Caco 2 cell permeability (Human colorectal carcinoma), Caco 2 cell model has been recommended as a reliable in vitro model for the prediction of oral drug absorption.

hERG - *in vitro* Human ether-a-go-go related gene (hERG) channel inhibition, The hERG channel inhibition assay is a highly sensitive measurement which will identify compounds exhibiting cardiotoxicity related to hERG inhibition *in vivo*. It is important to note, however that not all compounds which inhibit hERG activity *in vitro* will proceed to cause cardiotoxicity *in vivo*.

TPSA – Topological molecular polar surface area (PSA) is a very useful parameter for prediction of drug transport properties. Polar surface area is defined as a sum of surfaces of polar atoms (usually oxygens, nitrogens and attached hydrogens) in a molecule. This parameter has been shown to correlate very well with the human intestinal absorption, Caco-2 monolayers permeability, and blood-brain barrier penetration.

The bioactivity scores of the synthesized complexes were calculated for different parameters such as binding to G protein-coupled receptor (GPCR) ligand, ion channel modulator, kinase inhibition, nuclear receptor, protease inhibition, and enzyme inhibitor. If it is between −5.0 and 0.0, then the complex is moderately active, and if the bioactivity score is less than −5.0, then it is inactive.

IP - Intraperitoneal route of administration.

IV - Intravenous route of administration.

Oral - Oral route of administration.

SC - Subcutaneous route of administration.

LD50 - The amount of substance given at one time that resulted in death in 50% (1/2) of a group of experimental animals.

The Acute Rodent Toxicity Classification of Chemicals by the OECD Project consists of four categories. There are five acute toxicity classes in Globally Harmonised System (GHS). Acute rodent toxicity class 1 represents the most severe toxicity, with higher numbers indicating lower toxicity.

Table S2. Sequences of the primers used for quantitative RT-PCR.

Gene	Name	Primer
agrA	**Quorum-sensing regulator A**	Forward 5'-TGA TAA TCC TTA TGA GGT GCT T-3'<br>Reverse 5'-CAC TGT GAC TCG TAA CGA AAA-3'
aur	**Zinc metalloproteinase aureolysin**	Forward 5'-ACC GTG TGT TAA TTC GTG TGC TA-3'<br>Reverse 5'-ATG GTC GCA CAT TCA CAA GTT T-3'
icaA	**Intercellular adhesion A**	Forward 5′-TGA ACC GCT TGC CAT GTG-3'<br>Reverse 5′-CAC GCG TTG CTT CCA AAG A-3'
hla	**α-Hemolysin**	Forward 5'-CGG CAC ATT TGC ACC AAT AAG GC-3'<br>Reverse 5'-GGT TTA GCC TGG CCT TCA GC-3'
nuc1	**Nuclease**	Forward 5'-CAC CTG AAA CAA AGC ATC CTA A-3'<br>Reverse 5'-TAT ACG CTA AGC CAC GTC CAT-3'
RNAIII	**Transcriptional regulator**	Forward 5'-ATC GAC ACA GTG AAC AAA TTC AC-3'<br>Forward 5'-CTC TAC TAG CAA ATG TTA CTC AC-3'
saeR	**Response regulator**	Forward 5'-GCC TTA ACT TTA GGT GCA GAT GAC TAT GTC-3'<br>Forward 5'-CGA CAG TTG TTC AAC TGG TTG ATG ATG G-3'
sarA	**Transcriptional regulator**	Forward 5'-GAG TTG TTA TCA ATG GTC-3'<br>Reverse 5'-GTT TGC TTC AGT GAT TCG-3'
seb	**Enterotoxin B**	Forward 5'-TGT TCG GGT ATT TGA AGA TGG -3'<br>Reverse 5'-CGT TTC ATA AGG CGA GTT GTT-3'
sigB	**RNA Polymerase sigma factor**	Forward 5'-AAG TGA TTC GTA AGG ACG TCT-3'<br>Reverse 5'-TCG ATA ACT ATA ACC AAA GCC T-3'
spa	**Protein A**	Forward 5'-ACC AGA AAC TGG TGA AGA AAA TCC-3'<br>Reverse 5'-TAA CGC TGC ACC TAA GGC TAA TG-3'
16S rRNA	**A component of ribosomes**	Forward 5'-TGT TTG ACG ATG TTT GAG CA-3'<br>Reverse 5'-CCT TCC TCC AGT TCA GAT GC -3'
